# Identification of inflammatory protein biomarkers for predicting the different subtype of adult with tuberculosis: an Olink proteomic study

**DOI:** 10.1007/s00011-025-02020-9

**Published:** 2025-04-01

**Authors:** Yunlin Song, Buzukela Abuduaini, Xinting Yang, Jiyuan Zhang, Guirong Wang, Xiaobo Lu

**Affiliations:** 1 Department of Intensive Care Unit, The First Affiliated Hospital of Xinjiang Medical University, Urumqi, 830054 China; 2 Department of Intensive Care Unit, State Key Laboratory of Pathogenesis, Prevention and Treatment of High Incidence Diseases in Central Asia, The First Affiliated Hospital of Xinjiang Medical University, 393 South Li Yu Shan Road, Urumqi, 830054 Xinjiang China; 3 Tuberculosis Department, Beijing Chest Hospital, Capital Medical University, Beijing, 101149 China; 4 First Clinical Institute of Xinjiang Medical University, Urumqi, 830054 Xinjiang China; 5 Department of Clinical Laboratory, Beijing Chest Hospital, Capital Medical University, Beijing, 101149 China; 6 Center of Infection, The First Affiliated Hospital of Xinjiang Medical University, 393 South Li Yu Shan Road, Urumqi, 830054 Xinjiang China

**Keywords:** Biomarker, Inflammation, *Tuberculosis*, Olink proteomics

## Abstract

**Objective:**

This study aimed to identify the potential inflammatory molecular biomarkers that could be utilized for the early prediction of different subtypes of *tuberculosis* (TB) in adults.

**Methods:**

Plasma samples were obtained from a cohort of adults diagnosed with 48 cases of active TB, including drug-susceptible TB (S-TB, n = 28), multidrug-resistant TB (R-TB, n = 20), latent TB infection (LTBI, n = 20), as well as a control group of healthy individuals without any infection (HC, n = 20). The expression level of 92 inflammatory-related proteins was detected by using the high-throughput Olink proteomics platform.

**Results:**

There were 47 inflammatory proteins showing a significant difference (p < 0.05) among TB, LTBI, and HC groups, and 7 of them differed significantly between HC and LTBI groups, 43 proteins differed considerably between LTBI and TB groups, and overall, CXCL10 and TGF-alpha proteins differed substantially among the three groups which could be used as potential diagnostic biomarkers. Furthermore, SCF demonstrates remarkable discriminatory power in distinguishing TB from LTBI, with an area under the curve (AUC) score of 0.920. SLAMF1 has emerged as the top predictor for distinguishing Sputum Culture-Negative from positive *tuberculosis* cases, with an AUC of 0.779. The Correlation analyses showed various relationships among co-differentiated proteins. In LTBI versus HC, TGF-alpha and CXCL10 had a strong positive correlation. In non-severe versus severe TB, CXCL10 and CXCL9, as well as TNF and CCL3, were strongly positively correlated, while IL-6 and SCF had a negative correlation. These co-differentiated proteins were found to be enriched in various biological processes and molecular functions related to immune regulation and signaling pathways, such as the p53 signaling pathway, the TNF signaling pathway, and the NF-kappa B signaling pathway, highlighting the complex interplay of these proteins in the immune response to TB infection.

**Conclusion:**

Inflammation-related proteins exhibited distinct expression profiles in various conditions of TB. These proteins are intercorrelated and involve the pathogenesis of tuberculosis by activating diverse immune cells and promoting the secretion of pro-inflammatory cytokines. Their functions influence cellular phenotypes, which play a crucial regulatory role in the interaction between the host and *Mycobacterium tuberculosis*. These findings suggest that these proteins are potential disease prevention and treatment targets.

**Supplementary Information:**

The online version contains supplementary material available at 10.1007/s00011-025-02020-9.

## Introduction

*Tuberculosis*, a contagious disease caused by *Mycobacterium tuberculosis* (*M*. *tuberculosis)*, continues to present a substantial global health burden, with an estimated annual incidence of 10 million cases, rendering it a prominent contributor to morbidity and mortality on a worldwide scale [1]. The disease manifests in two distinct forms: active *tuberculosis*, which presents clinical symptoms, and latent *tuberculosis* infection (LTBI), where individuals have an immune response to the bacteria but show no symptoms. The risk of progressing from LTBI to ATB is notably higher in the first five years post-infection, particularly for children and those with compromised immune systems [2]. Early detection of LTBI is essential for effective prevention and management strategies [3].

Traditional diagnostic methods for *tuberculosis*, such as sputum smear microscopy, suffer from limited sensitivity and specificity, especially in cases of extrapulmonary TB or low bacillary loads. The gold standard, mycobacterial culture, is time-consuming and requires specialized facilities, making it less accessible in resource-limited settings. While faster, Molecular diagnostic tests are often expensive and do not always differentiate between latent and active infections, nor do they effectively detect antimicrobial resistance [4]. To address these challenges, there is an urgent need for the development of rapid, cost-effective, and user-friendly diagnostic methods that can be effectively deployed in the field, ensuring quick isolation of infected individuals and monitoring the efficacy of treatments.

Next-generation sequencing (NGS) technology offers a swift and cost-effective approach to DNA sequencing, facilitating the identification of drug-resistance mutations in the M. *tuberculosis* genome [5]. However, the high costs of implementing and maintaining NGS and the requirements for robust internet infrastructure and cloud computing may limit its application in low- and middle-income countries, necessitating the development of more low-cost diagnosis methods and affordable diagnostic solutions [6]. Understanding the pathophysiology of TB, especially in its early stages, is challenging to diagnose or differentiate from TB and LTBI. Standardized TB management is key to understanding its mechanisms, identifying biomarkers, and optimizing prevention, diagnosis, and treatment. This is vital given the growing epidemic of drug-resistant TB [7]. Olink proteomics is known for its high specificity and sensitivity in protein detection, requiring minimal sample volumes and being compatible with various sample types. It benefits targeted biomarker discovery and clinical diagnostics, where precision and sample conservation are key [8, 9].

The immune environment is crucial in the development of TB, influencing disease progression [10]. The Mycobacterium ensures its survival by intricately modulating the metabolic landscapes and immune responses within its host [11, 12]. Moreover, Proteins are key indicators of genetic and environmental disease risks, reflecting biological processes and changes in the body. Understanding protein-disease relationships can help identify unique biological signatures linked to different health states and disease progress [13–15]. This result suggests that immune and inflammation-related biomarkers may play a pivotal role in the progression of TB. To test this, we used healthy individuals or LTBI patients as control groups to compare with TB cases. Utilizing the Olink technology, an antibody-based proximity extension assay platform, we aimed to screen for circulating inflammatory biomarkers associated with TB in adults. This approach is intended to provide a more effective means of diagnosing TB at its earliest stages, thereby improving patient outcomes and strategically handling this significant global health challenge.

## Methods

### Patient recruitment

Between February 2022 and May 2023, adult individuals suspected of having TB were recruited prospectively at Beijing Chest Hospital (Beijing, China). Peripheral blood specimens were collected from these individuals. The inclusion criteria for the health control (HC) group were as follows: (1) no clinical signs of TB, (2) no abnormalities on X-ray or plain chest radiographs, and (3) the IGRA test was negative. The inclusion criteria for latent tuberculosis infection (LTBI) were [16]: (1) no clinical signs of TB, (2) no abnormalities on X-ray or plain chest radiographs, and (3) the IGRA test was positive. The inclusion criteria for active tuberculosis (TB) were: (1) With bacteriological evidence by any culture or Xpert from sputum; (2) Administered anti-TB drug for ≤ 3 Days in the past 6 months;(3) Excluding other chronic and acute conditions, including autoimmune diseases, malignant tumors, immunosuppressive therapy, and pregnancy.

Patients diagnosed with active tuberculosis (TB) are categorized into distinct subgroups based on the presence of multidrug-resistant *tuberculosis* (R-TB) versus drug-susceptible tuberculosis (S-TB), as well as the severity of the disease. The multidrug-resistant tuberculosis (R-TB) was defined as TB resistant to at least both isoniazid (INH) and rifampicin (RIF). Drug-susceptible TB (S-TB): A bacteriologically confirmed or clinically diagnosed case of TB without evidence of infection with strains resistant to isoniazid and rifampicin. Non-severe pulmonary TB: A form of TB defined as intrathoracic lymph node TB without airway obstruction; uncomplicated TB pleural effusion; or paucibacillary, non-cavitary disease confined to one lobe of the lungs and without a miliary pattern. Severe (or advanced) pulmonary TB disease: The presence of bilateral cavitary disease or extensive parenchymal damage on chest radiography (CXR). The Ethics Committee of Beijing Chest Hospital approved this study (Ethical approval No. YNLX-2022–006). Informed consent was obtained in writing from each participant.

## Specimen collection

A blood sample of 200 µl, anticoagulated with heparin, was collected within 12 h of the diagnosis of TB or LTBI, along with corresponding age-matched samples from the HC group. In cases where blood transfusion or surgical intervention was deemed necessary, the blood samples were procured before administering these treatment modalities. The plasma was separated by centrifugation (1000 g, 5 min) at 4 °C and stored at − 80 °C freezer.

## Olink assay

**Fig. 1:**
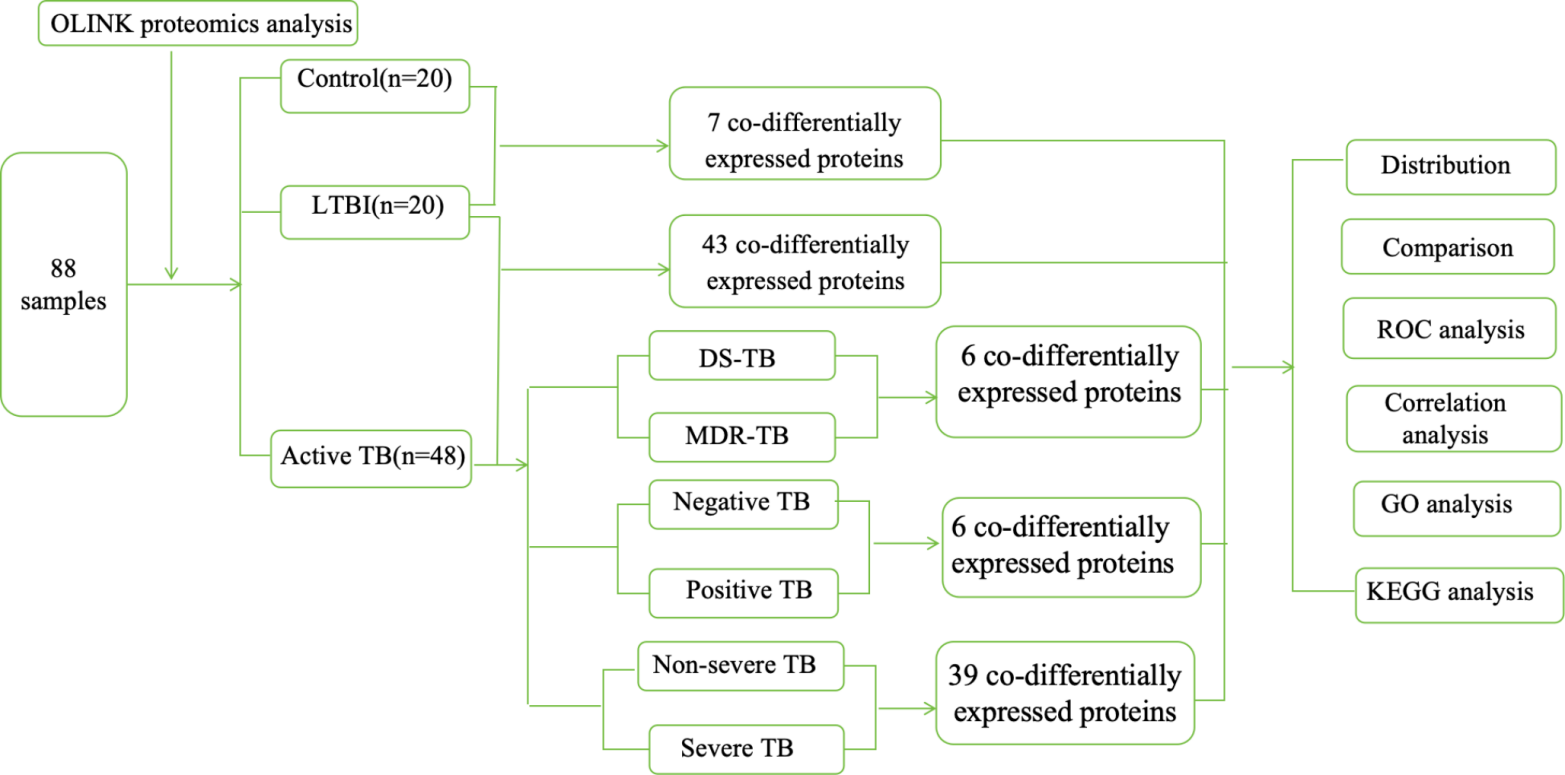
Flow diagram demonstrating the study design. The study utilized OLINK technology to detect inflammatory biomarkers in individuals classified with active tuberculosis (TB), latent tuberculosis infection (LTBI), and healthy controls (HC)

The protein levels were quantified utilizing the Olink inflammation panel (OLINK Proteomics AB, Uppsala, Sweden) according to the manufacturer's recommended instructions. The Proximity Extension Assay (PEA) technology employed in the OLINK protocol has been extensively documented [17]. The resulting Ct-data is then quality-controlled and normalized using a set of internal and external controls. The final assay results were presented in Normalized Protein expression (NPX) values, serving as an arbitrary unit on a log2-scale where increased values correlate positively with heightened protein expression levels. The flow chart of the study and the characteristics of proteins detected by Olink in active TB, LTBI, and HC groups (Fig. 1).

## Statistics analysis

The mean ± standard deviation was used to present normally distributed data, while the median (interquartile range, IQR) was used for non-parametric data. Qualitative data were summarized using percentages. Demographic data, clinical characteristics, and inflammation protein concentrations were compared among groups using appropriate statistical tests such as ANOVA, t test，Chi-square, Fisher exact, Mann–Whitney U, and Kruskal–Wallis tests. All inflammation protein concentrations were logarithm (2)-transformed for analysis. Univariate analysis was used to examine the correlation between levels of inflammation proteins and the risk of *tuberculosis,* comparing them with healthy control and latent *tuberculosi*s groups. ROC was used to determine the predictive accuracy of the nomogram model. Discrimination capacities were assessed by calculating the area under the receiver-operating characteristic curve (AUC), and the optimal cutoff value was determined based on the Youden index. Statistical analysis was conducted using R statistical software and SPSS 23.0 software (IBM, Armonk, USA). Principal component analysis (PCA) was performed using the ggplot2 package, version 3.3.5. A significance level of *p* < 0.05 was considered statistically significant. In figures, ****P < 0.0001, ***P < 0.001, **P < 0.01, and *P < 0.05.

## Results

### Population characteristics

This study included a total of 88 participants (Table 1), comprising 20 healthy controls (HC), 20 individuals with latent tuberculosis infection (LTBI), and 48 patients with active pulmonary tuberculosis (TB). Among those diagnosed with TB, patients were further stratified into subgroups based on the presence of multidrug-resistant *tuberculosis* (R-TB) versus drug-susceptible *tuberculosis* (S-TB), as well as the severity of the disease. There was no statistically significant difference between the three groups regarding age and gender. The IGRA test results show a significant statistical difference between the HC and LTBI groups. The IGRA (Interferon-Gamma Release Assay) is an in vitro test for detecting Mycobacterium tuberculosis infection. The QuantiFERON-TB Gold Plus (QFT®-Plus) is used for this testing. The Negative Result is that the IFN-gamma difference between the TB antigen and Nil tubes is less than 0.35 IU/ml. The Positive Result is that the IFN-gamma difference is greater than or equal to 0.35 IU/ml. Results should be interpreted alongside the patient's medical history, imaging studies, and clinical symptoms to assess for LTBI. There were no significant differences in the basic characteristics between the DS-TB and MDR-TB groups, except for D-Dimer (DD) and serum creatinine in the DS-TB group compared to the MDR-TB group (Table 2).

**Table 1:** Population Demographic data and baseline characteristic

Characteristics	HC (n = 20)	LTBI (n = 20)	TB (n = 48)	P value
Gender, *n*(%)				0.606
Male	11(55.00)	9(45.00)	28(58.30)	
Female	9(45.00)	11(55.00)	20(41.70)	
Age(years)	32.00 (26.25, 46.75)	44.50 (39.75, 52.75)	44.00 (29.25, 59.75)	0.091
IGRA test(IU/ml)	0 (0, 0)	1.509 (0.957, 4.710)	–	< 0.001

**Table 2:** Common Laboratory Values for S-TB and R-TB Populations

Variable	TB		P value
	S-TB (*n* = 28)	R-TB (*n* = 20)	
White blood cell	7.81 ± 4.27	7.76 ± 3.55	0.968
Hemoglobin	107.86 ± 24.00	113.45 ± 24.67	0.435
Platelet	298.50 ± 99.95	265.75 ± 119.73	0.308
Lymphocyte	1.13 ± 0.57	3.37 ± 7.48	0.195
Monocyte	0.48 ± 0.24	0.70 ± 0.61	0.093
Neutrophil	6.10 ± 4.32	10.21 ± 18.54	0.262
Albumin	12.86 ± 8.64	21.15 ± 32.12	0.273
Total bilirubin	11.19 ± 5.70	10.98 ± 3.98	0.886
serum creatinine	49.31 ± 14.96	64.08 ± 25.74	***0.016***
LDH	159.00 (137.25, 219.25)	144.00 (131.50, 172.00)	0.143
CRP	44.18(8.03, 73.04)	15.72(0.76, 55.07)	0.094
PCT	0.04(0.02, 0.14)	0.03(0.02, 0.08)	0.718
NT-proBNP	619.70 (421.25, 1744.75)	339.05 (66.90, 1439.20)	0.102
PH	7.43(7.41, 7.46)	7.45(7.44, 7.46)	0.242
PaCO_2_	37.00(34.50, 41.50)	42.00(35.00, 54.25)	0.138
PaO_2_	100.62 ± 24.87	123.86 ± 52.28	0.140
D-Dimer	1.70 ± 1.46	0.95 ± 0.90	***0.040***
NLR	4.02(1.84, 11.65)	3.78(2.43, 8.62)	0.707
**Smear**, *n*(%)			0.843
negative	12(42.9)	8(40.0)	
positive	16(57.1)	12(60.0)	
**severity of TB**, *n*(%)			0.692
non-severe	17(60.7)	11(55.0)	
severe	11(39.3)	9(45.0)	

CRP:C-reactiveprotein; PCT:Procalcitonin; NLR:Neutrophil-to-LymphocyteRatio; If P value is less than 0.05, it is expressed in bold and italic format

### Plasma protein profiling and PCA analysis: identifying differential expression and potential biomarkers in various subtypes of* tuberculosis*

**Fig. 2:**
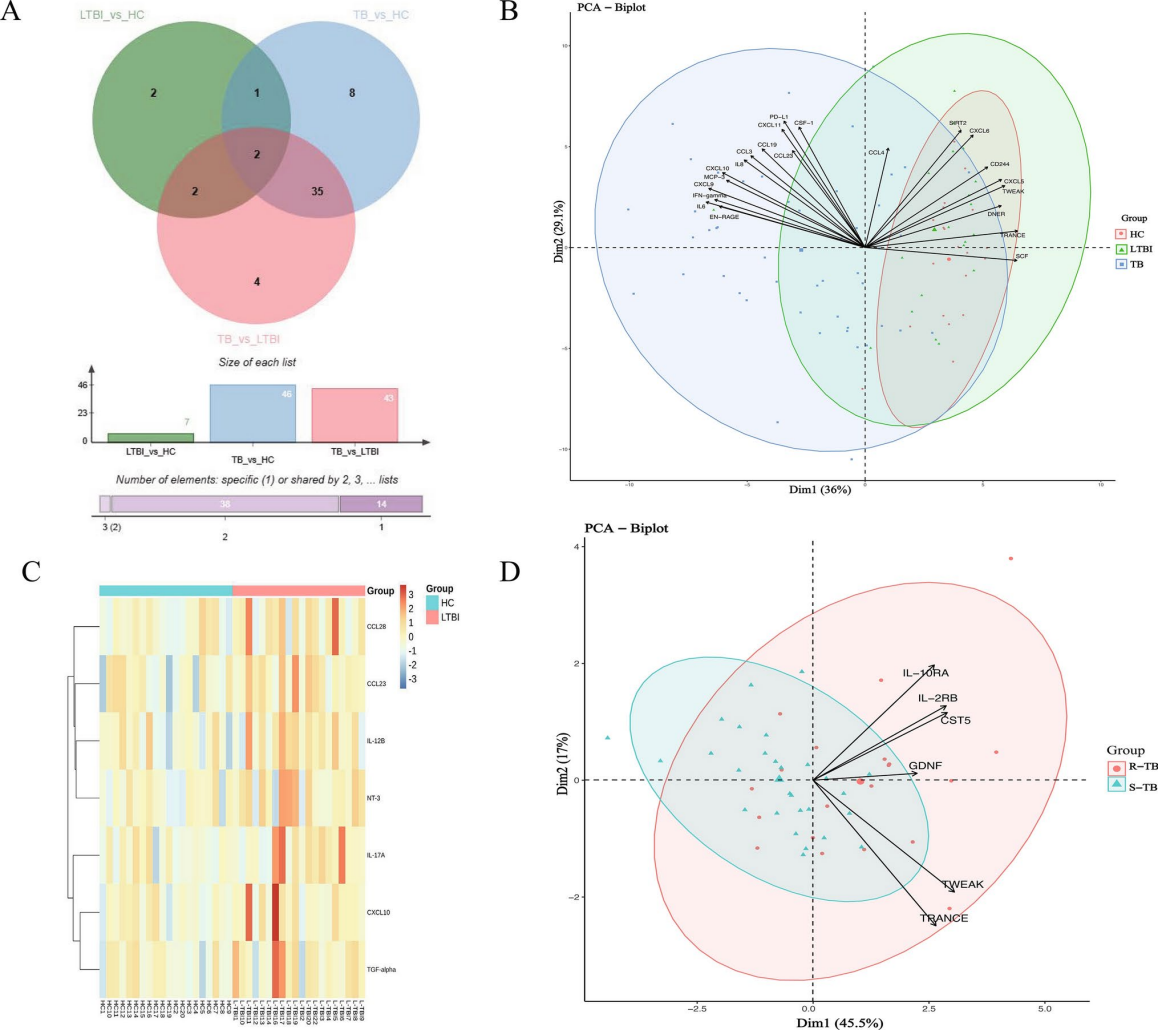
The characteristics of proteins detected by OLINK in HC, LTBI, and TB groups. (**A**) Wayne diagram showing the common and unique proteins in HC, LTBI, and TB groups. (**B**) Principal component analysis (PCA) based on 92 HC LTBI and TB proteins. (**C**) The heatmap analysis in individuals with hypercalcemia (HC) and latent tuberculosis infection (LTBI). (**D**) Principal component analysis (PCA) based on 92 proteins in S-TB and R-TB groups

All 92 plasma proteins were identified in the three groups, and 7 of them differed significantly between HC and LTBI groups, 43 proteins differed significantly between In the LTBI and TB groups, 46 proteins differed significantly between the TB and HC groups (Fig. 2A). Principal component analysis (PCA) revealed partial distinctions between HC and TB (Fig. 2B). The heatmap analysis shows partial differentiation in inflammation-related protein expression between HC and LTBI (Fig. 2C), as well as between multidrug-resistant *tuberculosis* (R-TB) and drug-sensitive *tuberculosis* (S-TB) (Fig. 2D). CXCL10, SCF, and TRANCE were the main contributors to variability in Dim1 (36.0%), while PDL-1 and CXCL11 were most important in Dim2 (29.1%). TRANCE and TWEAK were the critical factors in the variability among R-TB in Dim1 (45.5%).

## Comparative proteomic analysis: differential expression and diagnostic markers in various subtypes of *tuberculosis*

**Fig. 3:**
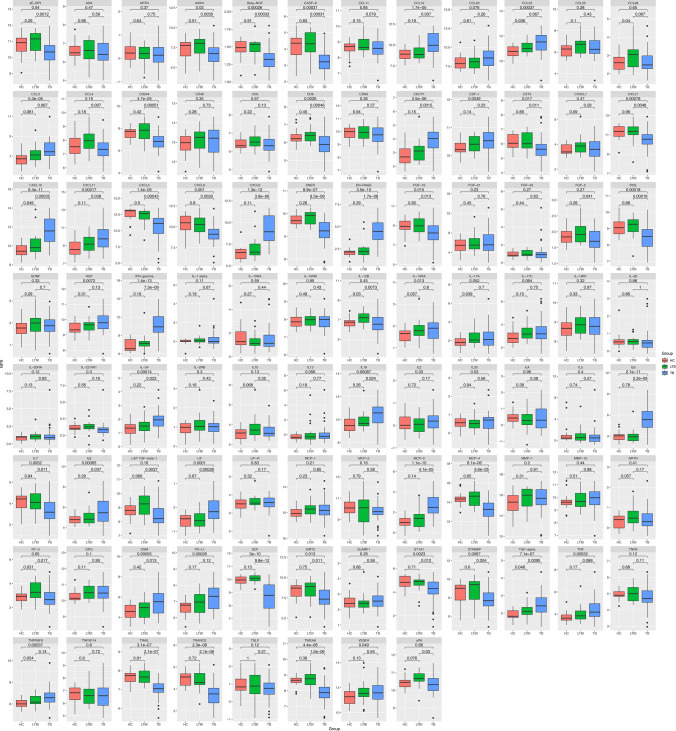
Box plot showing the expression level of differential proteins among these three groups. NPX (normalized protein expression) is OLINK’s arbitrary unit in Log2 scale It was calculated based on Ct values, and data pre-processing (normalization) was performed to minimize both intra- and inter-assay variation, *p < 0.05, **p < 0.01, ***p < 0.001

In comparing the active TB and HC groups, 46 differentially expressed proteins were identified. Compared to the HC group, IL-6, CXCL9, CXCL10, IFN-gamma, EN-RAGE, and MCP-3 were significantly elevated in active *tuberculosis*, while TRANCE, TWEAK, SCF, and TRAIL were notably decreased (Fig. 3).

Seven inflammatory proteins, including CCL23, CCL28, CXCL10, NT-3, IL-12B, IL-17A, and TGF-alpha, can potentially distinguish LTBI from HC groups. All seven proteins remained significantly different after statistical adjustments. Similarly, 43 inflammatory proteins can effectively distinguish TB from LTBI groups. Among them, the expression of IL-6, IFN-γ, and EN-RAGE were higher in TB. In contrast, the expression of SCF (Stem cell factor), DNER (Delta and Notch-like epidermal growth factor-related receptor (DNER), and TRAIL (TNF-related apoptosis-inducing ligand) proteins were lower in LTBI (Fig. 3).

CXCL10 and TGF-alpha showed statistically significant differences in expression levels between all pairwise comparisons of the three groups (HC, LTBI, and TB) and when comparing all three groups collectively. This indicates that CXCL10 and TGF-alpha proteins differed significantly among the three groups, which could be used as potential diagnostic biomarkers (Table 3).

**Table 3:** The diagnostic significance of TGF-alpha and CXCL-10 in differentiating between HC, LTBI, and TB

Variable	AUC: HC-LTBI	AUC: LTBI-TB	AUC: HC-TB
TGF-alpha	0.682	0.676	0.775
CXCL-10	0.650	0.760	0.815

## Identification and diagnostic potential of inflammatory proteins in R-TB, S-TB, LTBI

**Fig. 4:**
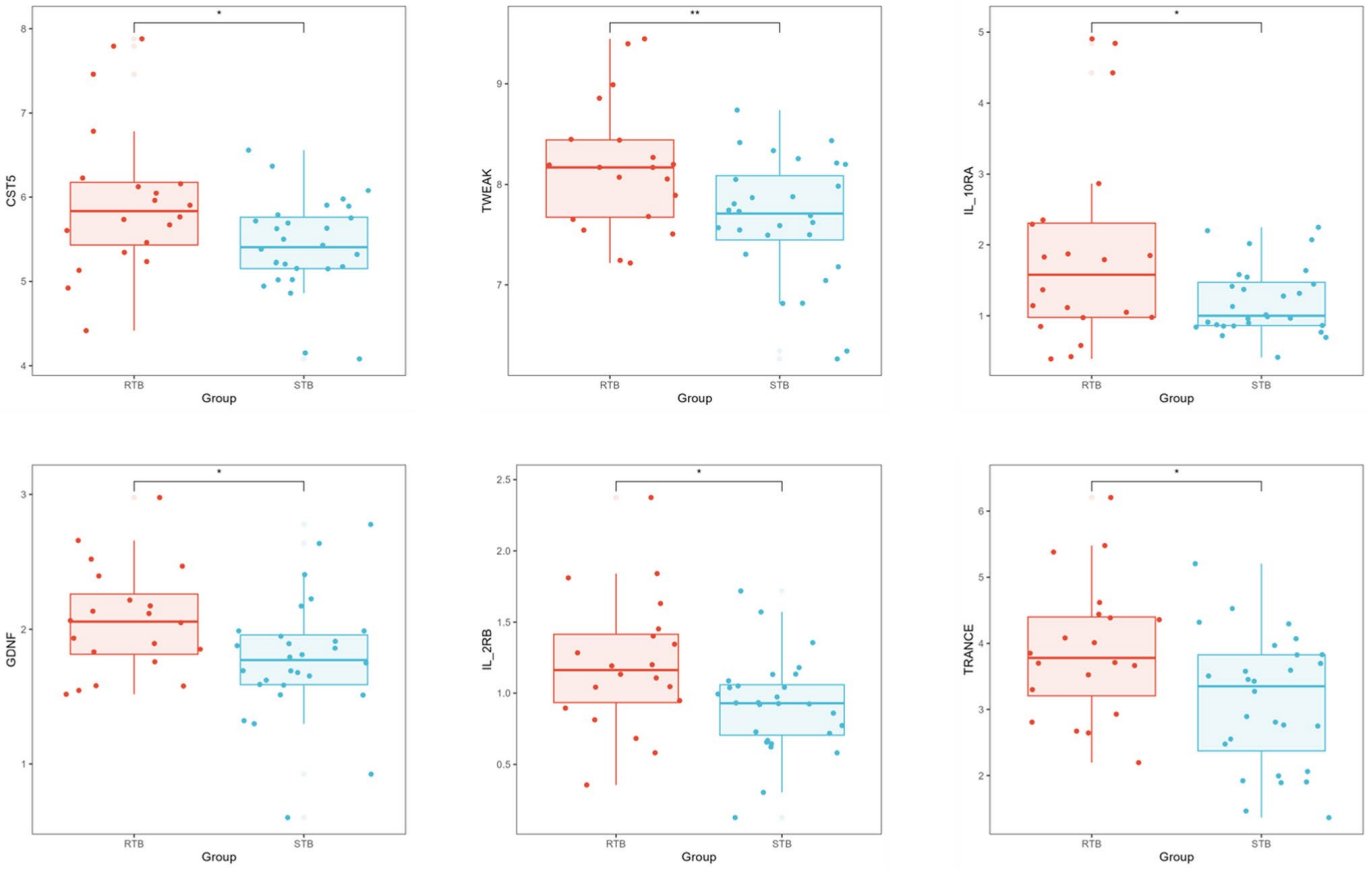
The co-differentially expressed proteins between the R-TB and S-TB Box plot showing the differences in co-differential proteins between the R-TB and S-TB groups. The horizontal axis represents the groups, and the vertical axis represents the protein expression levels by NPX. NPX is normalized protein expression, OLINK’s arbitrary unit in the Log2 scale. It is calculated from Ct values, and data pre-processing (normalization) is performed to minimize both intra- and inter-assay variation, *p < 0.05, **p < 0.01, ***p < 0.001.

Six proteins were differentially expressed in the comparative analysis between the Multi-Drug-Resistant *Tuberculosis* (R-TB) and Drug-Sensitive *Tuberculosis* (S-TB) groups. Specifically, IL-2RB, GDNF, CST5, TRANCE, TWEAK, and IL10RA levels were significantly reduced in the R-TB group compared to the S-TB group (Fig. 4).

**Fig. 5:**
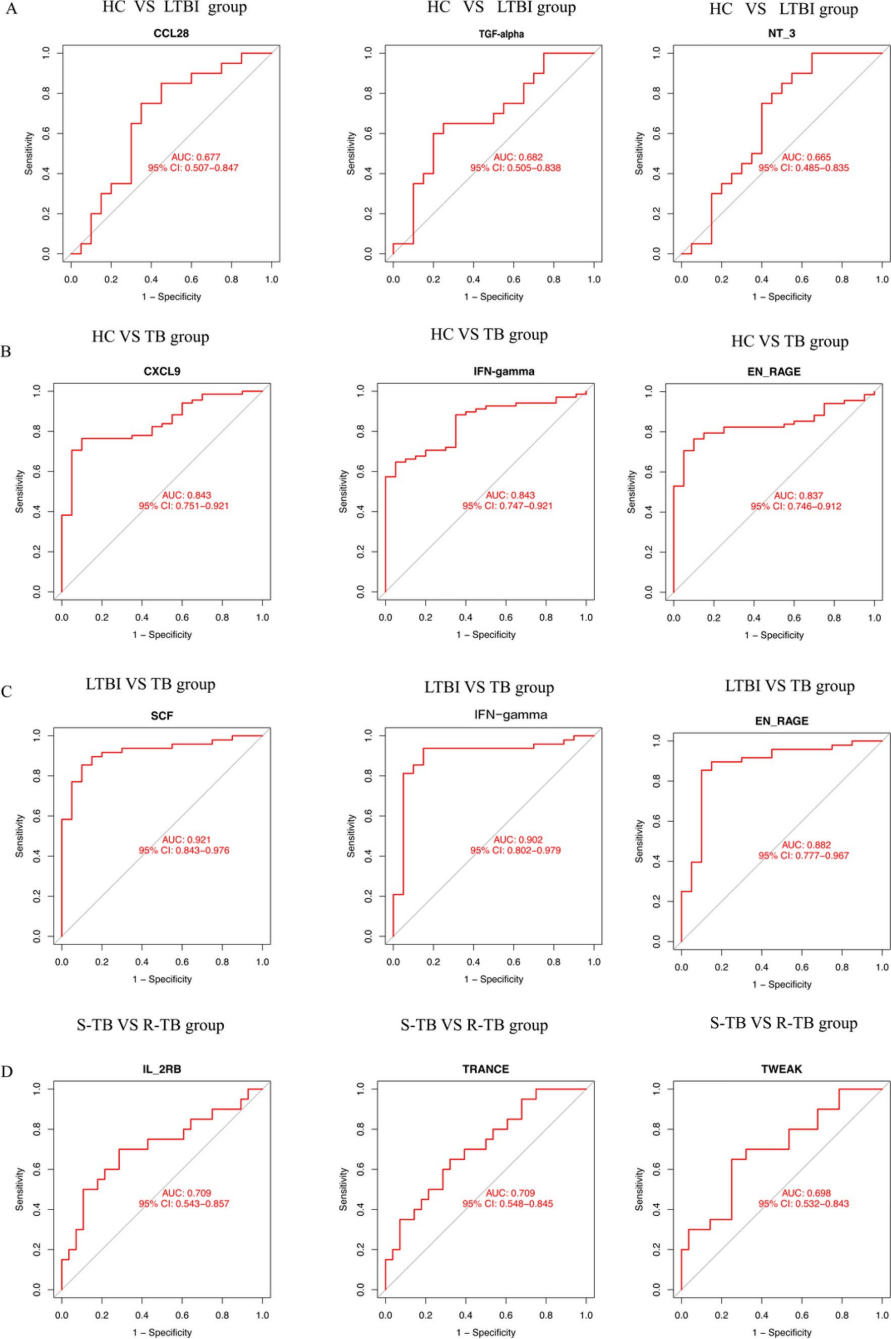
ROC curve of proteins in three groups. (**A**) Diagnostic efficacy of co-expressed proteins in distinguishing LTBI from HC. OR = odds ratio. (**B**) ROC curve analysis of CXCL9, IFN-alpha, EN-RAGE in HC and TB group. (**C**) ROC curve analysis of SCF, IFN-alpha, EN-RAGE in LTBI and TB group. (**D**) Characteristics of AUC of co-differential proteins in S-TB and R-TB group. AUC: Area under the curve

In evaluating the diagnostic value for distinguishing between LTBI and Healthy HC, the biomarkers CCL28 (AUC = 0.677), TGF-alpha (AUC = 0.682), and NT-3 (AUC = 0.665) exhibited low predictive capabilities (Fig. 5A). On the other hand, CXCL9 (AUC = 0.843), IFN-gamma (AUC = 0.843), and EN-RAGE (AUC = 0.837) showed good diagnostic value when differentiating between active TB and HC (Fig. 5B). These proteins indicate a stronger inflammatory response and immune activation in ATB patients than healthy individuals. Furthermore, SCF (AUC = 0.921), IFN-gamma (AUC = 0.902), and EN-RAGE (AUC = 0.882) displayed superior diagnostic value for distinguishing between LTBI and active TB. This highlights the potential of these proteins as biomarkers to identify the transition from a latent to an active state of the disease (Fig. 5C). In contrast, IL-2RB and TRANCE, which are inflammatory proteins, demonstrated moderate predictive power in distinguishing R-TB from S-TB, with their respective area under the curve (AUC) values being 0.709 (Fig. 5D).

However, the differential expression of specific inflammatory-related proteins holds promise as diagnostic biomarkers for various stages and severities of *tuberculosis*. The study underscores the significance of cytokine and chemokine dysregulation in the progression of the disease, as indicated by the AUC values in Fig. 4. These findings could contribute to a better understanding of TB pathogenesis and aid in developing more accurate diagnostic tools.

## Identification and diagnostic potential of inflammatory proteins in sputum culture-negative vs culture-positive pulmonary *tuberculosis*

**Fig. 6:**
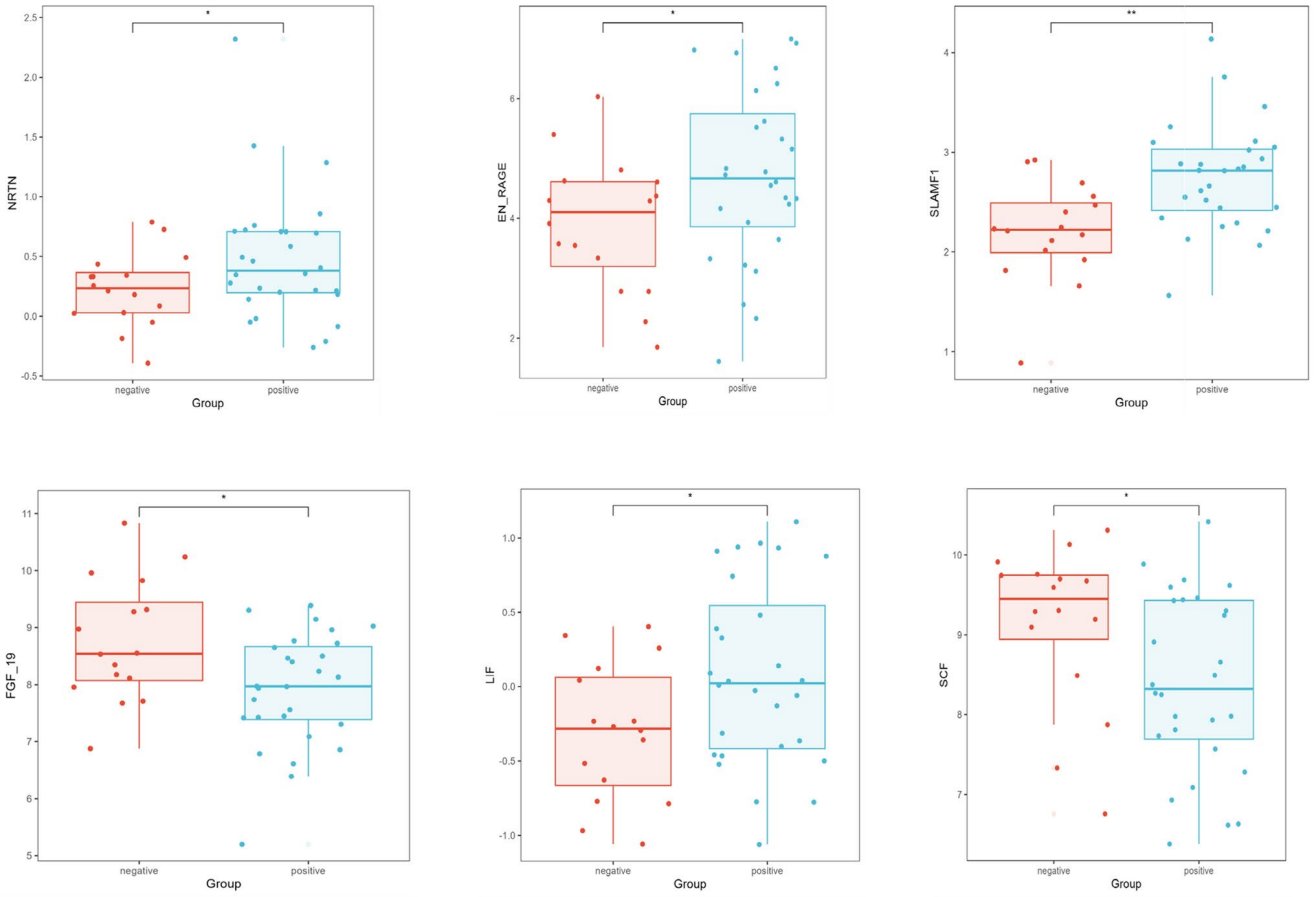
The co-differentially expressed proteins between the negative tuberculosis and positive TB. Box plot showing the differences in co-differential proteins between the negative tuberculosis and positive TB groups. The horizontal axis represents the groups, and the vertical axis represents the protein expression levels by NPX. NPX is normalized protein expression, OLINK’s arbitrary unit in the Log2 scale. It is calculated from Ct values, and data pre-processing (normalization) is performed to minimize both intra- and inter-assay variation, *p < 0.05, **p < 0.01, ***p < 0.001

**Fig. 7:**
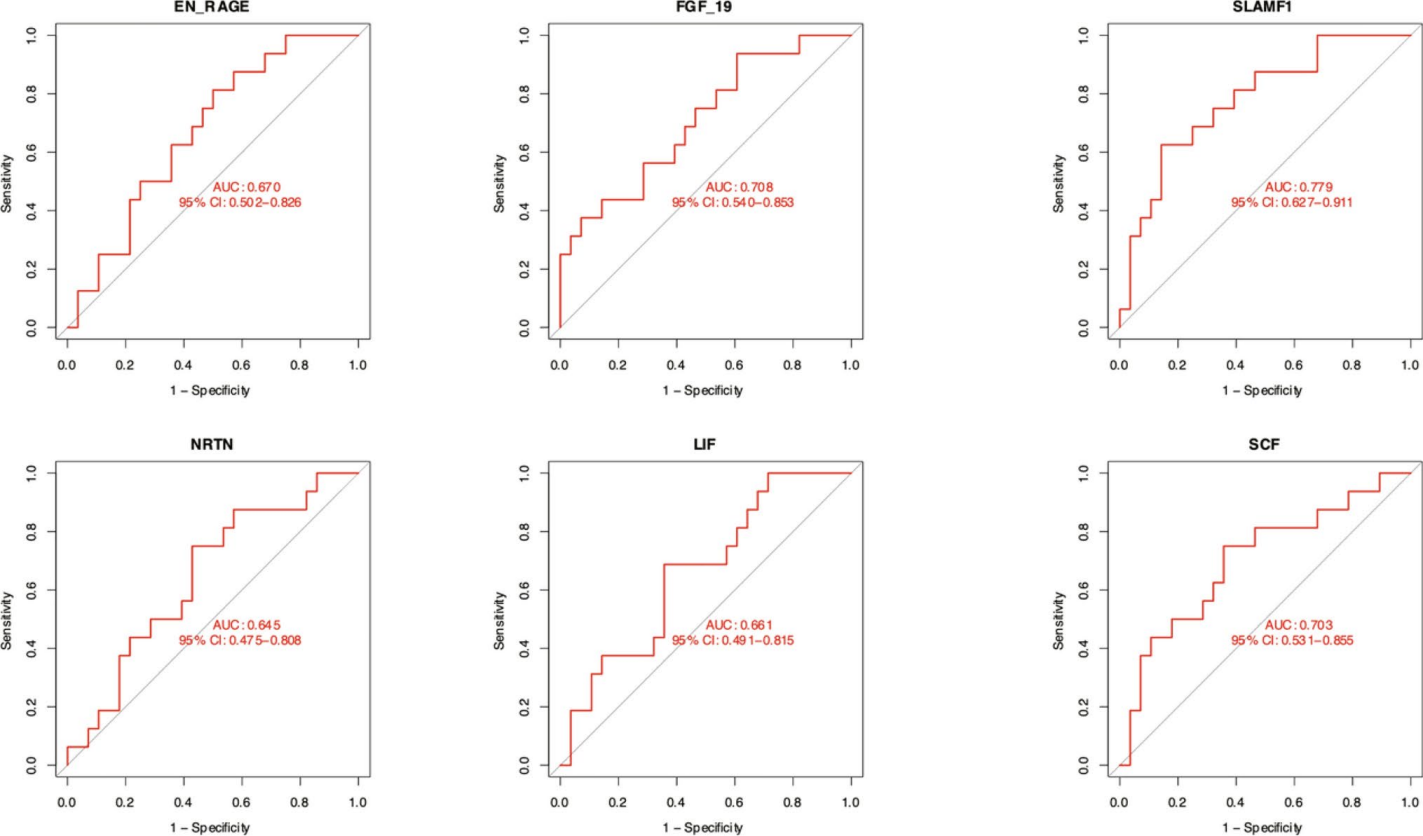
ROC curve of proteins in Sputum Culture-Negative vs Culture-Positive Pulmonary Tuberculosis

In this study, we examined the expression of inflammatory proteins in individuals with Sputum Culture-Negative Pulmonary *Tuberculosis* (Negative TB) and Sputum Culture-Positive Pulmonary *Tuberculosis* (Positive TB). We identified six inflammatory proteins—SLAMF1, SCF, LIF, NRTN, EN-RAGE, and FGF-19—that may be potential markers for distinguishing between the two groups. (Fig. 6). Of these proteins, SLAMF1 (AUC = 0.779) and FGF (AUC = 0.708) have potential value for differentiating negative TB from positive TB (Fig. 7).

## Identification and diagnostic potential of inflammatory proteins in severe pulmonary *tuberculosis*

**Fig. 8:**
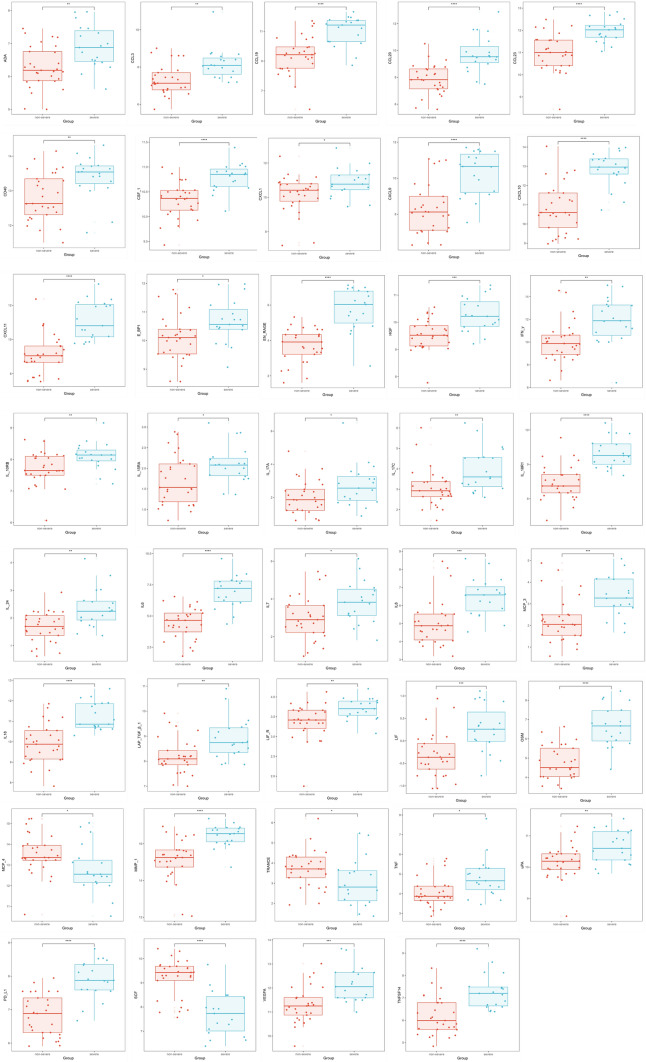
The co-differentially expressed proteins between the non-severe pulmonary TB and severe pulmonary TB. Box plot showing the differences in co-differential proteins between the non-severe pulmonary TB and severe pulmonary TB groups. The horizontal axis represents the groups, and the vertical axis represents the protein expression levels by NPX. NPX is normalized protein expression, Olink’s arbitrary unit in the Log2 scale. It is calculated from Ct values, and data pre-processing (normalization) is performed to minimize both intra- and inter-assay variation, *p < 0.05, **p < 0.01, ***p < 0.001

In our study, we have identified 39 inflammatory proteins that exhibit significant potential for distinguishing between different severities of pulmonary *tuberculosis*. Specifically, we observed a decrease in the expression of SCF (Stem Cell Factor), MCP-4 (Monocyte Chemoattractant Protein-4), and TRANCE (Tumor Necrosis Factor-Related Activation-Induced Cytokine) compared to non-severe cases. Conversely, the expression levels of the remaining 36 inflammatory proteins were found to be increased in severe cases (Fig. 8). This observation indicates a possible role these proteins play in the progression of the disease.

Furthermore, IL6, EN-RAGE, CXCL10, and PD-L1 demonstrated a notable diagnostic efficacy. The efficacy was determined by their high area under the curve (AUC) values, which exceeded 0.800, a threshold often used to indicate predictive solid performance in diagnostic tests. Although IL-6 is widely expressed during infections, in our study of patients with confirmed *tuberculosis*, we found that it assists in distinguishing the severity of the disease. IL-6, as a marker for severe *tuberculosis* patients, suggests that studying the formation and mechanisms of action of IL-6 is of significant importance for the prevention and treatment of *tuberculosis*. This suggests that the differential expression of specific inflammatory proteins can significantly indicate disease severity in pulmonary *tuberculosis*. Identifying these proteins, particularly those with high diagnostic efficacy, may contribute to developing more precise diagnostic tools and personalized treatment strategies for patients with severe pulmonary *tuberculosis* (Table 4).

**Table 4:** The diagnostic value of potential protein biomarkers between non-severe pulmonary TB and severe pulmonary TB

Name	Cutoff	Sensitivity	Specificity	AUC	95%CI	P values
IL6	0.521	0.850	0.926	0.933	0.864–1	8.85E-09
EN-RAGE	0.382	0.950	0.778	0.915	0.822–1	7.99E-08
CXCL10	0.506	0.850	0.889	0.907	0.817–0.998	3.37E-08
PD-L1	0.552	0.800	0.926	0.904	0.818–0.989	2.77E-07
OSM	0.474	0.800	0.852	0.891	0.798–0.983	4.31E-07
IL-18R1	0.361	0.950	0.742	0.889	0.797–0.981	6.87E-07
CXCL11	0.381	0.950	0.778	0.881	0.782–0.981	1.15E-06
CXCL9	0.328	0.950	0.741	0.881	0.782–0.981	1.16E-06
CCL20	0.493	0.750	0.926	0.880	0.779–0.98	3.18E-06
IL18	0.468	0.900	0.815	0.865	0.76–0.97	8.51E-06
CSF-1	0.462	0.800	0.852	0.857	0.748–0.967	1.92E-05
SCF	0.314	0.900	0.741	0.852	0.74–0.964	2.70E-06

## Correlation analysis of differentially expressed inflammatory proteins in various subtypes of *tuberculosis*

A clustered heat map was generated by further computing the Pearson correlation coefficients among pairwise differentially expressed proteins to investigate the correlation of inflammatory proteins within each group. The X and Y axes of the graph denote the names of these differentially expressed proteins, with red indicating a positive correlation and blue denoting a negative correlation. The intensity of the color reflects the strength of the correlation.

**Fig. 9:**
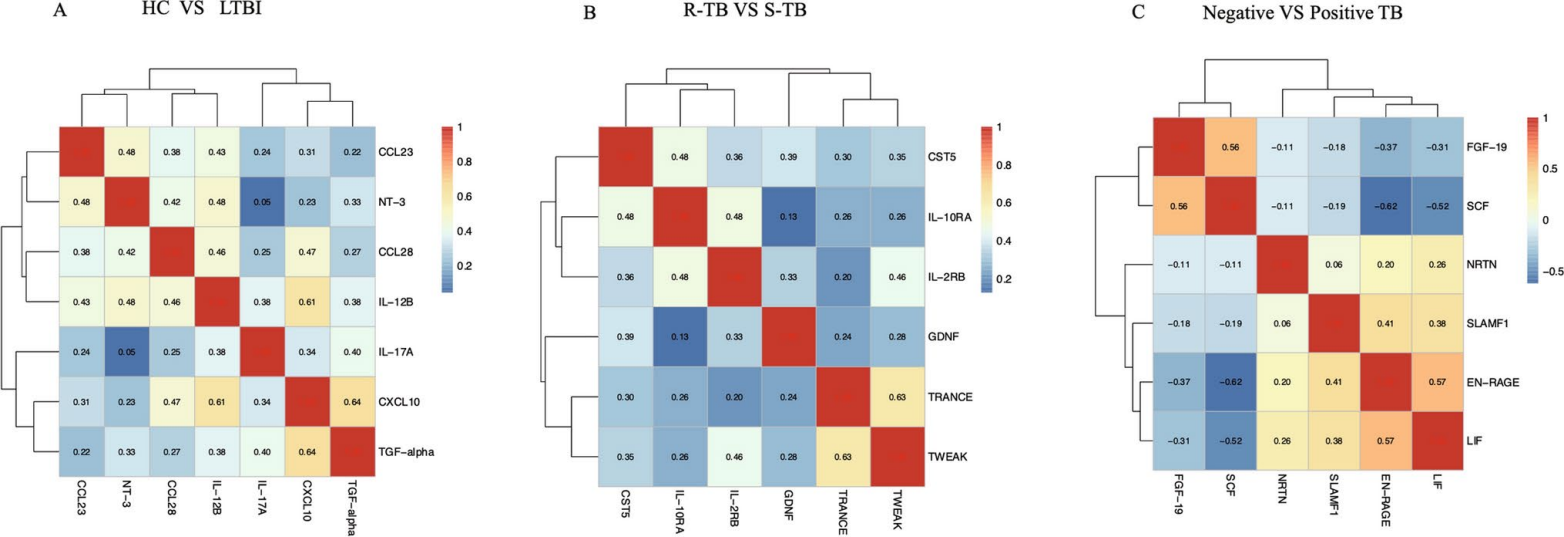
The Heatmap analysis of co-differentially expressed proteins among various groups. (**A**) The Heatmap analysis of inflammatory protein correlation in HC and LTBI group. (**B**) The Heatmap analysis of inflammatory protein correlation in S-TB and R-TB group. (**C**) The Heatmap analysis of inflammatory co-differentially expressed proteins in negative and positive TB groups

The co-differential proteins in individuals with LTBI and HC demonstrate a positive correlation between TGF-alpha and CXCL10, with a correlation coefficient of 0.638(Fig. 9A). Additionally, as shown in Fig. 9B, the co-differential proteins in S-TB and R-TB show a significant positive correlation for TRANCE and TWEAK (correlation coefficient = 0.6273). As shown in Fig. 9C, the co-differential proteins in negative and positive *tuberculosis* demonstrate a significant negative correlation for SCF and FGF 19 (correlation coefficient = -0.6184).

The co-differential proteins in individuals with TB and HC exhibit a strong positive correlation between SIRT2 and STAMBP and a negative correlation between SCF and IL-6 (Supplement Fig. 1A). Furthermore, in individuals with LTBI and TB, a positive correlation is observed between CXCL9 and CXCL10, as well as between STAMBP and CASP (Supplement Fig. 1B). As shown in Supplement Fig. 1C, the co-differential proteins in non-severe and severe *tuberculosis* exhibit a strong positive correlation for CXCL10 and CXCL9 (correlation coefficient = 0.920) and TNF and CCL3 (correlation coefficient = 0.924), as well as a negative correlation for IL-6 and SCF (correlation coefficient = -0.735). These findings indicate that inflammation-related proteins are intercorrelated.

## GO and KEGG analysis of co-differentially expressed proteins in various subtypes of *tuberculosis*

**Fig. 10:**
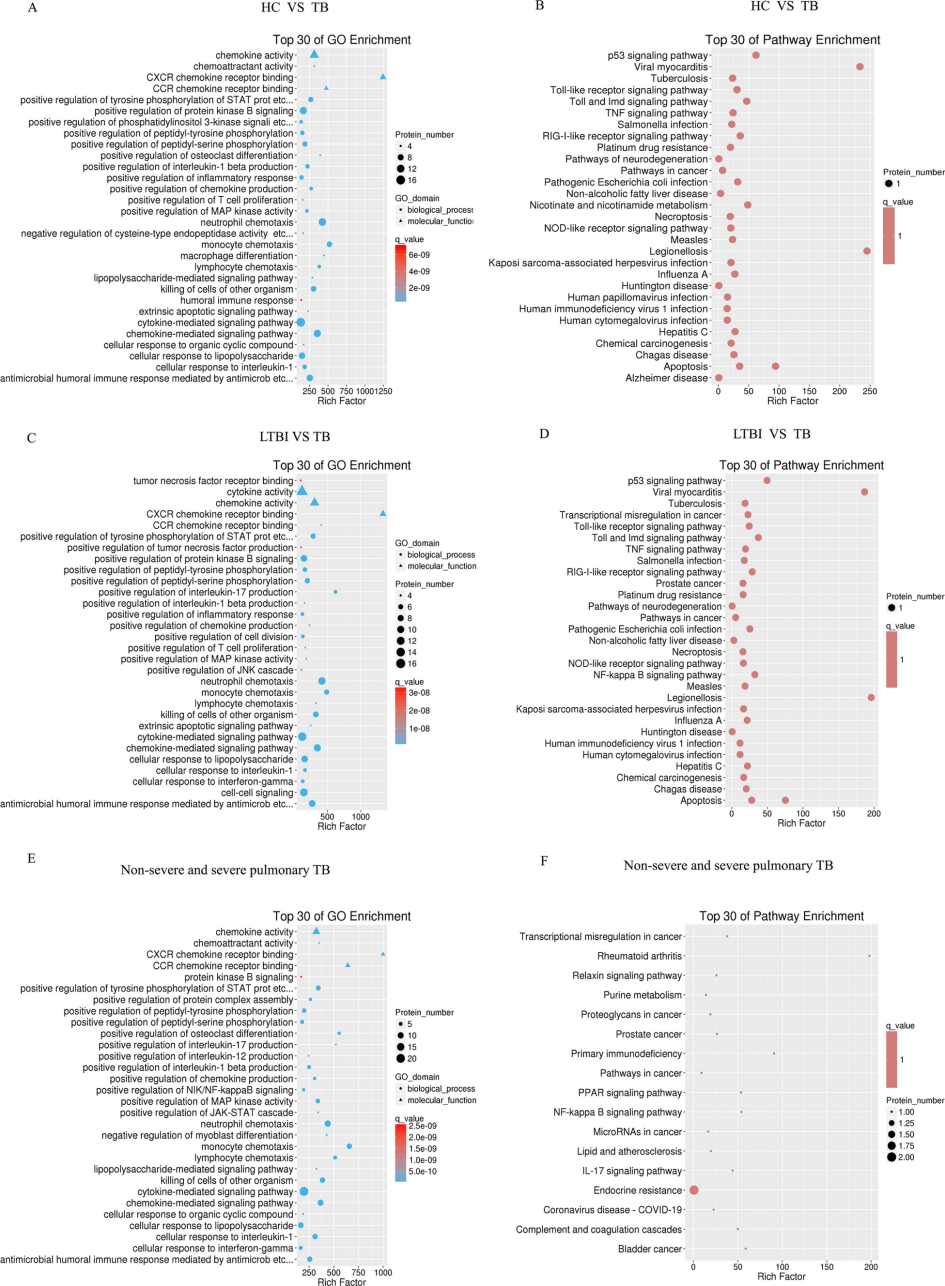
The analysis of Gene Ontology (GO) enrichment and Kyoto Encyclopedia of Genes and Genomes (KEGG) enrichment on co-differentially expressed proteins across distinct groups. (**A**) The analysis of GO enrichment on co-differentially expressed proteins HC and TB groups. (**B**) The analysis of KEGG enrichment on co-differentially expressed proteins HC and TB groups. (**C**) The analysis of GO enrichment on co-differentially expressed proteins LTBI and TB groups. (**D**)The analysis of KEGG enrichment on co-differentially expressed proteins in LTBI and TB groups. (**E**) The analysis of GO enrichment on co-differentially expressed proteins in non-severe and severe pulmonary TB groups. (**F**) The analysis of KEGG enrichment on co-differentially expressed proteins in non-severe and severe pulmonary TB groups

To confirm the role of co-differentially expressed proteins within three distinct groups, we conducted Gene Ontology (GO) and Kyoto Encyclopedia of Genes and Genomes (KEGG) enrichment analysis on these proteins. In the biological process (BP), co-differentially expressed proteins between HC and TB are primarily enriched in biological processes related to immune regulation, including positive regulation of interleukin-1 beta production, inflammatory response, lymphocyte chemotaxis, T cell proliferation, humoral immune response, macrophage differentiation, peptidyl-tyrosine and peptidyl-serine phosphorylation, MAP kinase activity, chemokine-mediated signaling pathway, and cellular response to lipopolysaccharide. In the molecular function (MF), co-differential expressed proteins are mainly enriched in chemokine activity and CXCR chemokine receptor binding (Fig. 10A). Furthermore, analysis of KEGG pathways indicates that these proteins are enriched in pathways such as the p53 signaling pathway, apoptosis Toll-like receptor signaling pathway, and TNF signaling pathway (Fig. 10B).

In the context of the biological process, the co-differentially expressed proteins between LTBI and TB are predominantly enriched in functions related to tumor necrosis factor receptor binding, positive regulation of JNK cascade, tumor necrosis factor production, peptidyl-tyrosine and peptidyl-serine phosphorylation, interleukin-17 production, cell proliferation, inflammatory response, interleukin-1 beta production, T cell proliferation, and chemokine production. In the molecular function (MF) context, co-differentially expressed proteins significantly enrich cytokine activity, chemokine activity, and CXCR chemokine receptor binding (Fig. 10C). Furthermore, KEGG pathway analysis reveals the enrichment of co-differentially expressed proteins in pathways such as the p53 signaling pathway, *Tuberculosis*, apoptosis, necroptosis, neurodegeneration pathway, TNF signaling pathway, and NF-kappa B signaling pathway (Fig. 10D).

In the biological process (BP), co-differential expressed proteins among Non-severe and severe TB are mainly enriched in protein kinase B signaling, positive regulation of interleukin-17 production, positive regulation of interleukin-12 production, cellular response to interferon-gamma, positive regulation of interleukin-1 beta production, positive regulation of chemokine production, positive regulation of NIK/NF-kappaB signaling, positive regulation of peptidyl-tyrosine and peptidyl-serine phosphorylation (Fig. 10E). The KEGG pathway analysis revealed that co-differentially expressed proteins exhibit enrichment in several pathways, including the PPAR signaling pathway, IL-17 signaling pathway, primary immunodeficiency, endocrine resistance, and NF-kappa B signaling pathway (Fig. 10F).

The outcomes of the GO and KEGG analyses of co-differentiated proteins across different groups highlight the significant involvement of immune regulation and cell signaling in the development of *tuberculosis*. Specifically, the dysregulation of cytokine and chemokine production and signaling emerges as a crucial factor in distinguishing between various stages and severities of the disease. These findings contribute to advancing knowledge regarding tuberculosis pathophysiology and can facilitate the development of targeted therapeutic interventions and diagnostic strategies.

## Discussion

Innate immune cells are the first to confront *M. tuberculosis*, significantly influencing the infection's progression. Dendritic cells (DCs) activate the adaptive immune response, while macrophages provide antimicrobial control and manage inflammation. The inflammatory response to *M. tuberculosis* can be a double-edged sword. Critical cytokines like TNF-alpha and IL-1 are necessary for protection, but excessive or insufficient levels can worsen the disease. Neutrophils, thought to control bacterial infections, also emerge as drivers of a harmful hyperinflammatory response. Understanding the balance between infection control and inflammation regulation is vital for developing effective host-targeted therapies [18, 19].

In this study, we utilized high-throughput Olink proteomics analysis technology to screen and identify precise and sensitive biomarkers for different subtypes of TB. Utilizing minimal sample requirements, we concurrently examined inflammatory proteins, identifying 47 proteins with significant expression among active TB, LTBI, and control healthy cases. Notably, the co-differentiated proteins were enriched in biological processes related to the proliferation and apoptosis of immune cells and chemokine-related activities. It is mainly enriched in the PPAR signaling pathway, IL-17 signaling pathway, primary immunodeficiency, and TNF signaling pathway. Identify the pathways and functions of co-differentiated proteins in TB subtypes to enhance our understanding of TB's clinical manifestations. These results indicate the importance of investigating how specific co-differentiated proteins may impact immune cell function, potentially decreasing inflammatory responses or enhancing the immune capabilities of patients for disease prevention and treatment.

LTBI screening is vital for identifying individuals at high risk of developing active *tuberculosis*, crucial for preventing disease progression and curbing its spread. It is essential in high-risk populations to reduce TB-related morbidity and mortality. Among these co-differentiated proteins, CXCL10 and TGF-alpha show potential as biomarkers for distinguishing between TB, LTBI, and the control healthy group. Recent observations show that CXCL10 is produced by antigen-presenting cells and activated macrophages during infection. This cytokine aids chemotaxis and leukocyte migration and may inhibit *Mycobacterium tuberculosis* replication [20–22]. CXCL10/IP-10 alone or combined with acute phase proteins or cytokines are proposed as markers of bacterial burden, LTB, and active TB discrimination [21, 23, 24]. Notably, CXCL10 levels significantly increase in patients with severe pulmonary tuberculosis. CXCL10, induced by interferon-gamma (IFN-gamma), is integral to the immune response against *tuberculosis*, as it aids in the recruitment of immune cells to the infection site and contributes to granuloma formation—a hallmark of TB pathology [24]. Previous research identified 24 parameters that were elevated exclusively in active TB, including TGF-alpha. Five parameters were increased in LTBI, specifically IL-5, IL-17F, IL-1, CCL20, and ICAM-1[25]. As observed, Influenza induces IL-8 and granulocyte–macrophage colony-stimulating factor (GM-CSF) secretion by human alveolar epithelial cells through recombinant human Hepatocyte growth factor (HGF)/c-Met and TGF-α/ epidermal growth factor receptor (EGFR) signaling [26]. The expression of TGF-alpha varies among individuals within a disease spectrum, and its expression trends still differ across various disease states. This indicates that the interaction between Mycobacterium tuberculosis and the host is heterogeneous and may depend on individual differences in innate immune responses.

Recently, research reported that chemokines CXCL10 and CXCL9 positively correlate and may serve as markers to distinguish between drug-resistant and drug-sensitive TB, enhancing disease stage differentiation [27]. In our study, the proteins CXCL10 and CXCL9 were not found to be distinct in R-TB and S-TB. CXCL10, a chemokine attracting immune cells to infection sites, is elevated in severe pulmonary disease patients’ serum and bronchoalveolar lavage fluid, positively correlating with disease severity [24, 28]. Additionally, Macrophages increase kynurenine (Kyn) production, activating the aryl hydrocarbon receptor (AhR). This activation upregulates cytokine signaling 3 (SOCS3) and inhibits the JAK-STAT1 pathway, reducing the secretion of the chemokines CXCL9 and CXCL10, vital for lung T-cell recruitment. In vivo mouse models show that knocking out AhR significantly enhances T-cell infiltration and activity, counteracting *Mycobacterium tuberculosis*-induced immunosuppression [29]. These results suggest that CXCL10 is associated with the progression of *Mycobacterium tuberculosis* infection and drug resistance through the regulation of T-cell immunity.

The co-expression of the protein IFN-gamma in LTBI and active TB indicates a significant increase in active TB and has substantial predictive value for active TB. Prior research has revealed that The CD5^+^ and CD10^+^ B cell subpopulations exhibit potential as biomarkers for distinguishing between LTBI and active TB. Specifically, LTBI is characterized by elevated levels of CD5^+^ B cells, which contribute to a cytokine-rich microenvironment containing IFN-gamma, IL-10, and IL-4. Conversely, active TB demonstrates an anti-inflammatory response only in stimulation with mycobacterial proteins or lipids [30]. CD5^+^ B cells may contribute to active TB progression via IFN-gamma. A recent study found that CD4^+^Foxp3^+^ cells have a time-dependent role in TB, with CCR4 crucially balancing IFN-gamma-mediated inflammation by managing these cells' influx and function. This suggests that targeting CD4^+^Foxp3^+^ cells or CCR4 could be a potential immunotherapy strategy, given TB's heterogeneity in immunocompetent adults [31]. Another protein, SCF, can differentiate between TB and LTBI. Various immune cells express the c-kit receptor and can be activated by SCF, contributing to fibrotic disorders. Eosinophils, for example, express c-kit and, when stimulated by SCF, produce pro-fibrotic cytokines like TGFβ and FGF, along with lipid mediators, proteases, and chemokines [32]. Integration of SCF and Leukotriene D4 (LTD4) signals may contribute to Mast cells (MCs) hyperplasia and hyper-reactivity during airway hyper-response and inflammation [33]. Its differential expression may indicate differences in inflammatory processes or the host’s attempt to control *M. tuberculosis*.

In this study, the PD-L1(Programmed Death Ligand-1), EN-RAGE (Extracellular Newly Identified RAGE-Binding Protein), and CXCL10 (Chemokine C-X-C Ligand 10) have significant diagnostic value for severe pulmonary TB. PD-L1 is a protein present on the surface of many immune cells [34]. It interacts with its receptor, PD-1, to regulate the immune system and prevent excessive inflammation [35]. In severe pulmonary TB, the bacterial load is high, triggering the expression of PD-L1 on immune cells as an immune evasion mechanism [36]. The other protein, EN-RAGE, also known as S100A12, is highly expressed in monocytes and neutrophils, acting as a DAMP to interact with RAGE [37, 38]. It triggers signal transduction through the NF-κB and MAPK pathways, enhances the expression of ICAM-1, VCAM-1, NF-κB, and TNF-alpha, and directly activates endothelial cells, mononuclear phagocytes, and lymphocytes. This activation leads to the synthesis and secretion of proinflammatory cytokines, recruiting leukocytes to the inflammatory site, and, ultimately, the onset of inflammation [39]. It can activate monocytes through the TLR4 pathway, enhance monocyte migration, and upregulate proinflammatory factors such as IL-1β, IL-6, and IL-8 [40]. These findings suggest that they contribute to the struggle between the host and pathogen in both normal and inflammatory conditions, making them potential targets for disease prevention and treatment [41].

SLAMF1 (Signaling Lymphocytic Activation Molecule Family Member 1) protein can identify early or paucibacillary tuberculosis in Sputum Culture-Negative Pulmonary Tuberculosis patients, serving as an essential inflammatory factor that predicts negative pulmonary tuberculosis. SLAMF1 has been identified as a potential biomarker for Sputum Culture-Negative TB prediction due to its association with immune system functions and inflammatory response [42]. A previous study showed that SLAMF1 expression was reduced in active TB patients [43]. High SLAMF1 levels may indicate a normal immune response and lower TB risk. Therefore, SLAMF1 can predict sputum culture negative and positive TB status, a critical mechanism that needs further research.

The TRANCE (Tumor Necrosis Factor-Related Activation-Induced Cytokine) and IL-2RB (Interleukin-2 Receptor Beta) immune-related molecules [44, 45] exhibit significant predictive value for R-TB, with levels of TRANCE and IL-2RB being higher in R-TB than S-TB. Reports indicate that TRANCE can be a biomarker for diagnosing and assessing tuberculosis [46]. Previous research has shown that TRANCE expressed on activated T cells promotes the survival of dendritic cells and modulates T helper cell responses to viral infections [47]. Growing evidence has demonstrated that TRANCE is preferentially expressed on the surface of activated CD4^+^Th1 cells and dendritic cells through binding to RANK and enhances IFN-γ secretion via a p38-dependent pathway [48]. IL-2RB deficient patients demonstrated decreased Treg cell frequency, skewing toward memory T cells, and lymphocytic infiltration into multiple tissues [49, 50]. These molecules are integral to activating and regulating T-cell immunity, vital for controlling and eliminating mycobacterial infections.

In this research, creatinine is higher in R-TB, and D-Dimer is higher in S-TB,

Mainly correlated with the S-TB patients who were relatively older R-TB, and previous studies reported D-dimer levels, lactate dehydrogenase, C-reactive protein, erythrocyte sedimentation rate, and creatinine kinase were positively correlated with patient age [51]. Notably, A previous study reported that Urinary neopterin/creatinine ratios are significantly higher in patients with active tuberculosis than in patients with latent infection and may be a significant predictor of active tuberculosis in patients with *M. tuberculosis* infection [52]. Furthermore, Elderly tuberculosis patients are at high risk for thrombosis and renal injury. These conditions must be closely monitored during disease progression to prevent thrombosis and protect renal function.

The findings of this study have significant implications, especially in the context of potential advancements in TB diagnosis and treatment. The discovery of IL-2RB, TGF-alpha, and other proteins as potential biomarkers can transform the approach to TB diagnosis. These biomarkers offer the possibility of developing more accurate diagnostic tools to distinguish between active TB, LTBI, and control groups with heightened sensitivity and specificity. Furthermore, the predictive value of immune-related molecules like TRANCE and IL-2RB for multi-drug-resistant tuberculosis offers a promising avenue for the early identification of at-risk patients and developing strategies to combat drug resistance. This could be crucial in the fight against the rising global threat of multi-drug-resistant tuberculosis. The potential of proteins like SLAMF1 to distinguish between positive and negative could lead to earlier disease detection, allowing for interventions before the onset of symptoms. Understanding the role of these proteins in the immune response to TB could open new avenues for targeted therapies that enhance or modulate the immune system's reaction to the disease. Moreover, the study's findings could be instrumental in developing prognostic models that assess treatment outcomes by monitoring changes in biomarker levels. This could provide clinicians with valuable insights into the efficacy of treatments and disease progression.

This study is subject to several limitations. Firstly, additional validation with a larger sample size is necessary to elucidate the role of differential proteins in predicting tuberculosis across various disease states. Secondly, ongoing monitoring of specific proteins in patients with similar conditions is essential to understand the potential mechanisms of action of these proteins in the disease. Future research endeavors will address these limitations and advance the identification of early diagnostic biomarkers.

In summary, the importance of co-differentially expressed proteins in diagnosing the severity or subtype of TB lies in their correlation with immune responses and inflammatory processes during TB infection. Nevertheless, it is imperative to underscore the need for further research to validate their diagnostic value and establish standardized cut-off thresholds for their clinical application.

## Supplementary Information

Below is the link to the electronic supplementary material.

Supplementary file1 (PDF 306 kb)

## Data Availability

No datasets were generated or analysed during the current study.

## Abbreviations

CCL3: C–C motif chemokine 3
IL-2RB: Interleukin-2 receptor subunit beta
CSF-1: Macrophage colony-stimulating factor1
NT-3: Neurotrophin-3
PD-L1: Programmed cell death 1 ligand 1
EN-RAGE: Extracellular Newly Identified RAGE-binding protein
SLAMF1: Signaling lymphocytic activation molecule
STAMPB: STAM-binding protein
SCF: Stem cell factor
TRANCE: TNF-related activation-induced cytokine
TRAIL: TNF-related apoptosis-inducing ligand
TGF-alpha: Transforming growth factor alpha
TWEAK: Tumor necrosis factor (Ligand) superfamily, member 12
TNF: Tumor necrosis factor
DAMP: Damage-Associated Molecular Pattern
RAGE: Receptor for Advanced Glycosylation End-products
MAPK: Mitogen-Activated Protein Kinase
ICAM-1: Intercellular Adhesion Molecule-1
VCAM-1: Vascular Cellular Adhesion Molecule-1
