# Supplementary material for: Identification of inflammatory protein biomarkers for predicting the different subtype of adult with tuberculosis: an Olink proteomic study
Source: Inflamm Res. 2025 Apr 1;74(1):60. doi: 10.1007/s00011-025-02020-9 (PMC11958430; doi:10.1007/s00011-025-02020-9)
Supplement: Supplementary file 1 — Supplementary file1 (PDF 306 kb) [file 11_2025_2020_MOESM1_ESM.pdf]

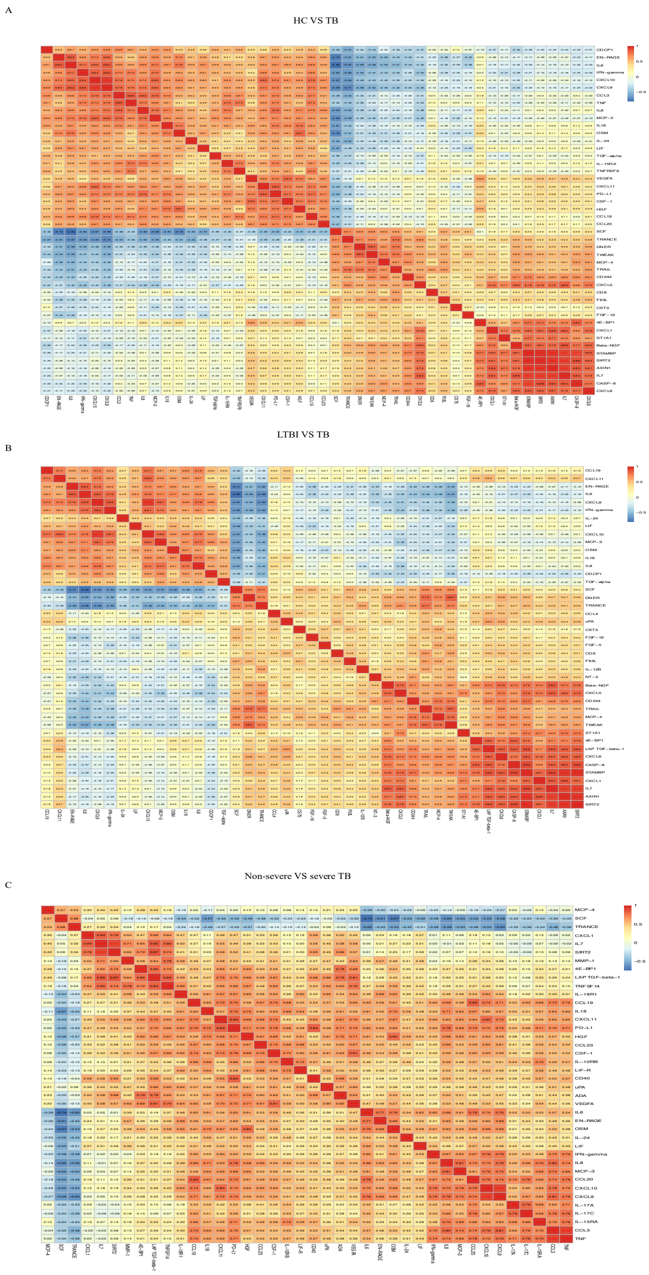

Supplement Figure 1. The Heatmap analysis of co-differentially expressed proteins among various groups. **(A)** The Heatmap analysis of inflammatory protein correlation in HC and TB group. (**B**) The Heatmap analysis of inflammatory protein correlation in LTBI and TB group. (**C**) The Heatmap analysis of inflammatory co-differentially expressed proteins in non-severe and severe TB groups.
